# Effect of foaming parameters on the physical and phytochemical properties of tomato powder

**DOI:** 10.1007/s10068-022-01125-9

**Published:** 2022-07-02

**Authors:** Eman Farid, Sabah Mounir, Eman Talaat, Sherif Elnemr, Hassan Siliha

**Affiliations:** Department of Food Science, Faculty of Agriculture, Zagazig University, Zagazig, 44519 Egypt

**Keywords:** Foam-mat-drying, Tomato powder, Soy protein isolate, Physical and phytochemical properties

## Abstract

The objective of this work was to study the effect of foaming parameters on the physical and phytochemical properties of tomato powder. A central composite rotatable design of experiments was defined with two parameters (concentration of soy protein isolate [SPI]: 1–5% and whipping time: 2–14 min) with 5 levels for each parameter. The foam was prepared by whipping tomato puree after adding SPI and dried in a thin layer (4 mm ± 1) at 50 °C. The obtained results showed the predominant effect of the concentration of SPI on the physical and phytochemical properties compared to whipping time. The powder prepared under foaming conditions of 5% SPI and whipping time of 8 min showed an increase of 97, 39, 62, and 46% in the total phenolics, total flavonoids, antioxidant activity, and porosity, respectively, while the bulk density decreased by about 25%.

## Introduction

Tomatoes (*Solanum lycopersicum*) belong to the Solanum genus of the Solanaceae family and are widely used in food preparations in fresh (salad) and dried states. Dried tomatoes can be used in the preparation of pizza and in various ready-to-eat meals as well. Tomatoes are rich in polyphenols such as flavonoids, carotenoids, and vitamins, which provide antioxidant, anti-mutagenic, anti-proliferative, and anti-inflammatory properties (Tan et al., 2010). Tomatoes are also an important source of dietary fibers, essential amino acids, and minerals such as Na, K, Mg, P, and Ca. Tomatoes have a relatively short shelf life due to their high moisture content (93–96%), causing a great loss during transportation, distribution, and storage. Therefore, the preservation of tomatoes is necessary to maintain their quality and to reduce the loss during the storage period. Drying is one of the oldest methods of food preservation and widely used to extend the storage period of tomatoes. However, the quality attributes of dried tomatoes greatly depend on the drying method used and on the drying conditions applied. Although sun drying is commonly used to dehydrate tomatoes because of the low costs, but it has some constraints like the long operation time, possible risk of environmental contamination, and the occurrence of enzymatic and non-enzymatic reactions, which may cause lowering quality attributes of sun-dried tomatoes (Lahsasni et al., 2004). As a result, hot air drying is used as an alternative method to sun drying due to its shorter drying time. But, this technique also has drawbacks such as high energy consumption and poor quality attributes in terms of sensory, nutritive, and functional properties. These drawbacks are related to the shrinkage phenomenon and texture compactness, which occur during conventional hot air drying, decreasing the effective moisture diffusivity (Yusufe et al., 2017). In order to overcome these drawbacks, hot air foam-mat-drying (FMD) could be used for the production of tomato powder as an alternative technique. Hot air FMD is an energy-efficient and cost-effective technique compared to other conventional drying techniques such as spray-drying and freeze-drying. This technique is based on the transformation of liquid or semi-liquid foods into stable foam which thereafter dried in a thin layer or sheet by hot air at relatively low temperatures. Foam is formed by the incorporation of a considerable amount of air bubbles which trapped into the foam structure during whipping for a given time after adding an appropriate foaming agent. The drying time is less than other techniques due to the open porous structure and the large specific surface area (exchange surface) exposed to drying air which accelerate moisture removal (Wilson et al., 2012). Therefore, the quality of the foamed powder is higher than that obtained by conventional hot air drying as a result of the short drying time and the relatively lower drying temperature. Few studies have been reported dealing with the effect of foaming parameters on the physical and phytochemical properties of FMD tomato powder (Hossain et al., 2021; Kadam et al., 2012). Similarly, no studies were reported on the use of soy protein isolate (SPI) as a foaming agent in the production of FMD tomato powder and also the studies that deal with the physical characteristics of tomato powder were observed. For these reasons, the objective of this work was to study the effect of the concentration of SPI as a foaming agent and the whipping time on the physical and phytochemical properties of hot air foam-mat- dried tomato powder.

## Materials and methods

### Raw materials

Fully ripe fleshy red tomato fruits were purchased from the local market (Zagazig, Sharkia government, Egypt). Tomato fruits (about 50 kg) were manually sorted and washed with running clean tap water. Inedible parts were manually removed, and rewashed with running clean tap water. In order to remove the skin of tomatoes, tomatoes were subjected to steam for 5 min until wrinkling the skin and cooled immediately under running clean tap water.

### Foaming agent

Spray-dried soy protein isolate powder (90% protein) was obtained from Agricultural Research Center (Dokki, Giza, Egypt). A 32% solution was prepared by dissolving SPI powder in distilled water with a ratio of 1:2 w/w (SPI: distilled water) ensuring a complete dissolution of SPI powder in the mixture. According to the experimental design, the final concentration of SPI was prepared using a 32% solution, which varied from 0.32% to 1.6% and defined according to preliminary trials. It was found that the foam density stated to increase after reaching the maximum decrease at a higher concentration than 1.6% of SPI. Moreover, the final concentration of SPI added in this study was close to that used by Brar et al. (2020) who optimized the FMD parameters of peaches using three different concentrations of SPI; 0.5%, 1%, and 1.5%.

### Experimental design and hot air drying

**Fig. 1:**
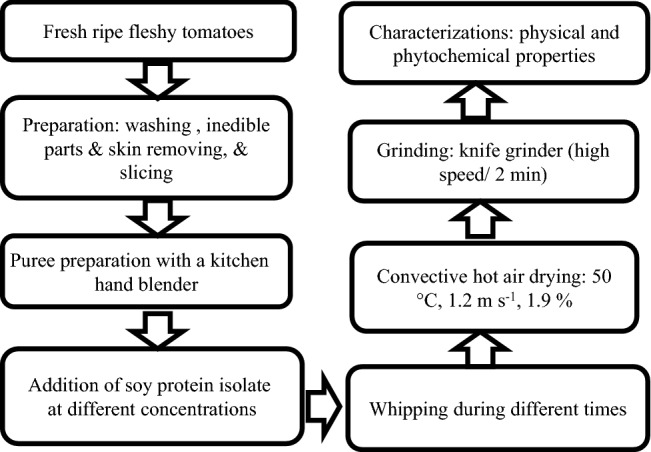
Protocol used for the production of hot air FMD tomato powder prepared with different concentrations of 32% SPI solution during different times of whipping

A central composite rotatable design of experiments was defined with two parameters [foaming agent concentration (*C*) and whipping time (*t*)] with 5 levels for each parameter representing 4 factor points, 4 star-points, and 5 replications of the center point (Table 1). Tomato fruits were crushed with an electrical hand blender (Braun Multi Quick 1 Hand Blender, 450 Watt, MQ 120, Poland) in order to obtain tomato puree. The puree was then mixed with a given concentration of SPI and whipped for a given time, using the same blender, according to the experimental design. The whipped mixture was subsequently spread in a thin layer (4 ± 1 mm as thickness) on a silicon sheet and dried at 50 °C with air characterizations of 1.2 m s^−1^ and 1.9% as velocity and relative humidity, respectively. Tomato slices (4 ± 1 mm thickness) were used for comparison; tomatoes were prepared as mentioned above, sliced, and then dried under the same conditions. The dried samples were ground using a kitchen knife grinder (Moulinex, 500 W) at high speed for 2 min at room temperature (30 ± 2 °C) and all characterizations were performed on < 160 µm tomato powder. The samples were referred to as HAD and FMD for conventional hot air dried sample (control sample) and hot air foam dried samples, respectively (Fig. 1).

**Table 1:** Experimental design for the production of hot air FMD tomato powder prepared with different concentrations of SPI during different times of whipping

C (%)	3	4	2	5	3	3	3	3	1	4	4	3	3
t (min)	4	14	12	8	8	8	2	8	8	12	4	8	8

*FMD* Foam-mat-dried tomato powder, *C* concentration of SPI (%) (Prepared from 32% solution), *t* whipping time (min)

### Assessments and characterizations

#### Bulk and intrinsic densities

The bulk density (ρ_bulk_) of tomato powder was determined according to Vasudevan et al. (2020). The method is based on measuring the volume occupied by 20 g of powder poured in a 50 mL measuring cylinder without tapping. The ratio between the mass and the volume is expressed as the bulk density of the powder (Kg/ m^3^).

The intrinsic density (ρ_intrinsic_) of tomato powder was determined according to the method described by Sørensen et al. (1978), but with a slight modification. The powder sample (1 g) was transferred into a 10 mL measuring cylinder with a glass stopper followed by adding 5 mL of petroleum ether. The measuring cylinder was the shaken to ensure the dispersion of the powder particles. The powder particles which present on the wall of measuring cylinder were rinsed down using a further 1 mL of petroleum ether. The total volume of petroleum ether (6 mL) with dispersed powder was recorded and the intrinsic density of powder was calculated according to Eq. 1:

1 $$\rho_{{{\text{intrinsic}}}} = \frac{{{\text{mass of powder}} \left( {1 g} \right)}}{{t{\text{otal volume of petroleum ether with dispersed powder}} \left( {{\text{mL}}} \right) - 6}}$$

where: 6 is the total volume of petroleum ether used in the measurement (mL).

#### Relative expansion ratio and porosity

The relative expansion ratio (ε_rel_) of FMD tomato powder was calculated according to Mounir (2015) using Eq. 2:

2 $$\varepsilon_{{{\text{rel}}}} = \frac{{\rho_{{{\text{HAD}}}} }}{{\rho_{{{\text{FMD}}}} }}$$

where: ρ_HAD_ is the bulk density of conventional hot air dried powder (HAD powder: control sample), ρ_FMD_ is the bulk density of hot air foam-mat-dried powder (FMD powder).

The porosity of tomato powder was calculated according to Mounir and Allaf (2017) using Eq. 3:

3 $${\text{Porosity}} =1-\left(\frac{{\rho }_{\text{bulk}}}{{\rho }_{\text{int}}}\right)$$

where: ρ_bulk_ is the bulk density of tomato powder, ρ_int_ is the intrinsic density of tomato powder.

#### Preparation of the methanolic extract

About 10 g of tomato powder were dissolved in 100 mL of methanol and filtered through Whatman No.1 filter paper (diameter 50 cm, pore size 11 µm). The residue was re-extracted with further 60 mL of methanol and filtered through Whatman No.1 filter paper. The filtrate was evaporated by rotary vacuum evaporator at 40 °C (Buchi Rotavapor R-124 Rotary Evaporator, Switzerland).

#### Total phenolic content

The total phenolic content (TPC) of the methanolic extract was quantified according to Elfalleh et al. (2009) using the Folin-Ciocalteu method. Briefly, 0.5 mL of methanolic extract was added to 0.5 mL of Folin-Ciocalteu reagent and vortexed for 3 min followed by adding 4 mL of Na2CO3 solution (1 M). The mixture was incubated at 45 °C for 5 min in darkness and at the end of this period, it was cooled in a cold water bath. The absorbance was read at 765 nm, using a spectrophotometer (Jenway 6705, UK), against a blank sample containing distilled water. The TPC was calculated on the basis of the calibration curve of Gallic acid and expressed as mg equivalents of Gallic acid per 100 g dry basis (mg GAE/100 g db).

#### Total flavonoid content

The aluminium chloride colorimetric technique was used for flavonoids quantification according to Elfalleh et al. (2009). Briefly, 1 mL of methanolic extract was added to 1 mL of 2% AlCl_3_ methanolic solution. The mixture was vortexed for 3 min and allowed to stand for 15 min at room temperature (30 ± 2 °C). The absorbance was read at 430 nm using a spectrophotometer (Jenway 6705, UK), against a blank sample containing distilled water. TFC was calculated on the basis of the calibration curve of quercetin and expressed as mg equivalents of quercetin per 100 g dry basis (mg QE/100 g db).

#### DPPH radical scavenging assay

The antioxidant activity of the methanolic extract was assessed by measuring their scavenging abilities to 2, 2-diphenyl-1-picrylhydrazyl stable radical. The DPPH assay was carried out as described by Miliauskas et al. (2004), but with slight modifications. Briefly, 2 mL of methanolic extract was mixed with 2 mL of DPPH methanolic solution (0.1 mM) and the mixture was kept at room temperature (30 ± 2 °C) for 30 min in darkness. The absorbance of the tested samples was read at 517 nm against a blank sample containing 2 mL DPPH and 2 mL methanol. The radical scavenging activity (%) was calculated by using the following formula:

4 $$\mathrm{Radical\, scavenging }\,(\mathrm{\%}) = \left[\frac{{Abs}_{\text{blank}}-{Abs}_{sample}}{{Abs}_{blank}}\right]\times 100$$

### Statistical analysis

The results were presented as mean value ± standard deviation of three replicates and statistically analyzed by ANOVA in order to identify the significant differences between the response variables at *p<0.05*  . The statistical analysis was limited to analyze only the findings of the total phenolic compounds owing to that the coefficient of variation (CV) was more than 10%. Moreover, response surface methodology (RSM) by Statgraphics plus (1994–4.1 version) was used to describe the effect of foaming parameters (*C*, *t*) on the responses including 3-D response surface graph. A correlation matrix was also carried out in order to describe the correlation between the foaming parameters (*C*, *t*), and each of the response variables.

## Results and discussion

### Bulk and intrinsic  densities

Bulk density (ρ_bulk_) is defined as a ratio between the mass of powder and its volume without tapping, including the interstitial air (air present between the particles), while the intrinsic density (ρ_intrinsic_) is the sum of the density of the solids (proteins, carbohydrates, fat, and minerals) and the amount of occluded air. Bulk density is a good indicator of structural changes which occur during the processing of foods and it may affect the flowability and the instantaneity properties of the powder. The low bulk density of a powder indicates its excellent flowability with no caking behavior (Carr, 1965) and good instant characteristics, due to the low values of both Carr index (CI) and Hausner ratio (HR).

The bulk density of HAD tomato powder was 752 kg m^−3^, while that of FMD powder was varied from 562 to 726 kg m^−3^, with a decrease of 25% for the powder prepared under foaming conditions of *C* = 5% and *t* = 8 min, compared to HAD powder (Table 2). On the other hand, the intrinsic density of HAD powder was 1503 kg m^−3^, while that of FMD powder ranged from 1900 to 2327 kg m^−3^, with an increase of about 55% for FMD powder prepared under foaming conditions of *C* = 5% and *t* = 8 min, compared to HAD powder (Table 2). The higher bulk density of HAD powder could be explained by the dense structure of the powder which resulted from the texture compactness owing to the shrinkage phenomenon (Mounir and Allaf, 2017). In contrast, the low bulk density of FMD powder may be related to the open porous structure which resulted from the incorporation of the air bubbles into the foam structure (Djaeni et al., 2015; Franco et al., 2016) as well as the opening of finer pores and the formation of new pores owing to the movement and the migration of the moisture from the product's core towards its external surface, during the drying operation (Sankat and Castaigne, 2004). While, the higher intrinsic density of FMD powder, compared to HAD one, may be due to the addition of SPI, which contributed to the increase in the protein content, leading to an increase in the total solids of FMD tomato powder.

**Table 2:** Physical and phytochemical properties of hot air FMD tomato powder prepared with different concentrations of SPI during different times of whipping

Foaming conditions	ρ_bulk_	ρ_int_	ε_rel_	Porosity	TPC	TFC	AOA
HAD (Control)	752 ± 2.5	1503 ± 2.3	100 ± 0.00	50.0 ± 0.1	422.3 ± 1.1^j^	227.3 ± 1.1	43.9 ± 0.2
C: 2%, t: 4 min	702 ± 1.5	1980 ± 1.0	107.1 ± 0.01	57.9 ± 0.1	513.9 ± 1.0^g^	264.0 ± 1.0	55.9 ± 0.2
C:3%, t: 14 min	596 ± 2.0	2139 ± 1.5	126.2 ± 0.00	67.7 ± 0.02	545.3 ± 1.2^f^	272.9 ± 1.1	56.3 ± 0.3
C: 2%, t: 12 min	671 ± 1.5	1985 ± 1.5	112.07 ± 0.01	60.6 ± 0.03	446.7 ± 1.3^hi^	251.9 ± 1.0	51.2 ± 0.3
C: 5%, t: 8 min	562 ± 1.0	2327 ± 1.5	133.81 ± 0.02	72.8 ± 0.1	829.8 ± 1.7^a^	316.6 ± 1.3	71.1 ± 0.2
C: 3%, t: 2 min	667 ± 1.1	2132 ± 1.8	112.74 ± 0.00	63.3 ± 0.04	607.2 ± 1.1^d^	284.8 ± 1.2	62.6 ± 0.3
C: 1%, t: 8 min	726 ± 1.1	1900 ± 1.1	103.58 ± 0.01	54.1 ± 0.03	445.7 ± 1.4^h^	252.4 ± 1.1	47.3 ± 0.3
C: 4%, t: 12 min	581 ± 1.0	2270 ± 1.1	129.43 ± 0.01	70.8 ± 0.03	717.0 ± 1.1^c^	285.2 ± 1.1	62.5 ± 0.2
C: 4%, t: 4 min	568 ± 1.4	2274 ± 1.0	132.39 ± 0.0	71.7 ± 0.03	751.0 ± 1.1^b^	293.1 ± 1.2	64.5 ± 0.3
C: 3%, t: 8 min	612 ± 1.2	2138 ± 1.8	122.88 ± 0.02	67.1 ± 0.04	595.0 ± 1.3^e^	281.6 ± 1.3	60.4 ± 0.3

*FMD* Foam-mat-dried tomato powder, *C* concentration of SPI (%), *t* whipping time (min), *ρ*_*bulk*_ bulk density (Kg/m^3^), *ρ*_*int*_ intrinsic density (Kg/m^3^), *ε*_*rel*_ relative expansion ratio (%), *TPC* total phenolic content (mg GAE/100 g db), *TFC* total flavonoid content (mg QE/100 g db), *AOA* antioxidant activity (%)

Superscript letters are statistically significantly different (*P* < 0.05)

**Fig. 2:**
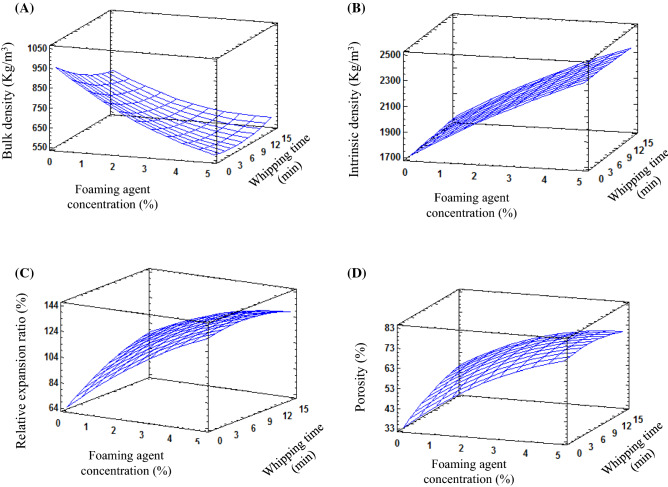
Response surface graph for physical properties of hot air FMD tomato powder prepared with different concentrations of 32% SPI solution during different times of whipping: (**A**) bulk density (Kg/m^3^), (**B**) intrinsic density (Kg/m^3^), (**C**) relative expansion ratio (%), and d) porosity (%)

The effect of parameters (*C, t*) on the bulk and intrinsic densities of FMD tomato powder is illustrated in Fig. 2. The bulk density showed a gradual decrease with an increase in the SPI concentration and the whipping time, reaching a certain extent and started to increase afterwards. This behavior could be explained by two phenomena; (1) weakening of air bubbles owing to the whipping for a long time, (2) increasing of total solids which in turn increase the mixture viscosity exceeding the limited viscosity at which more of air bubbles could be easily incorporated into the foam structure (Khamjae and Rojanakorn, 2018). On the contrary, the intrinsic density showed an increasing trend with an increase in SPI concentration, with an insignificant effect of whipping time. The increase in intrinsic density may be due to a large amount of occluded air within the powder particles and SPI’s addition.

### Relative expansion ratio and porosity

Many textural changes such as texture compactness and structure collapse are related to the shrinkage phenomenon, that occurs during the conventional hot air drying (Muñoz-López et al. 2018), which in turn decline the drying rate. Thus, the quality attributes in terms of nutritive, sensory, physical, and functional properties may be degraded owing to the long drying time. The relative expansion ratio and porosity were calculated to describe the invisible changes in the texture of HAD and FMD tomato powders and could be used as an indicator of external (interstitial air) and internal porosity (occluded air). The relative expansion ratio and porosity of tomato powders are shown in Table 2. The relative expansion ratio of HAD was 100%, while that of FMD tomato powder ranged from 103 to 133.8%, with an increase of about 34% for the powder prepared under foaming conditions of *C* = 5% and *t* = 8 min, compared to HAD powder. The porosity of HAD powder was found to be 50%, while that of FMD powder varied from 54.1% to 72.8%, with an increase of about 46% for the powder prepared under foaming conditions of *C* = 5% and *t* = 8 min, compared to HAD powder. The lower relative expansion ratio and porosity of HAD tomato powder may be due to the texture compactness and the more dense structure as a result of the shrinkage phenomenon that occurred during the conventional hot air drying. The effect of foaming parameters (*C*, *t*) on the relative expansion ratio and porosity of FMD tomato powder is illustrated in Fig. 2. A gradual increase in the relative expansion ratio and porosity was observed with increasing the concentration of SPI and the whipping time, reaching a certain extent, and started to decrease afterwards. The increase in the relative expansion ratio and porosity of FMD tomato powder may be related to the porous structure which resulted from (1) the incorporation of a considerable amount of air bubbles into the foam structure which in turn causes an accumulation of air within the particles making them more porous and less dense (Goula and Adamapoulos, 2008), and (2) the formation of new pores as a result of moisture migration and movement during FMD process as previously mentioned (Sankat and Castaigne, 2004). Low bulk density powder with a porous structure shows better reconstitution properties than high bulk density powder with a dense structure (Kandasamy et al., 2019). Similar results were obtained by Sankat and Castaigne (2004) and Sharada (2013). Sankat and Castaigne (2004) reported that banana powder prepared with soy protein was brittle with a porous structure compared to non-foamed powder.

### Total phenolic and flavonoid contents

**Fig. 3:**
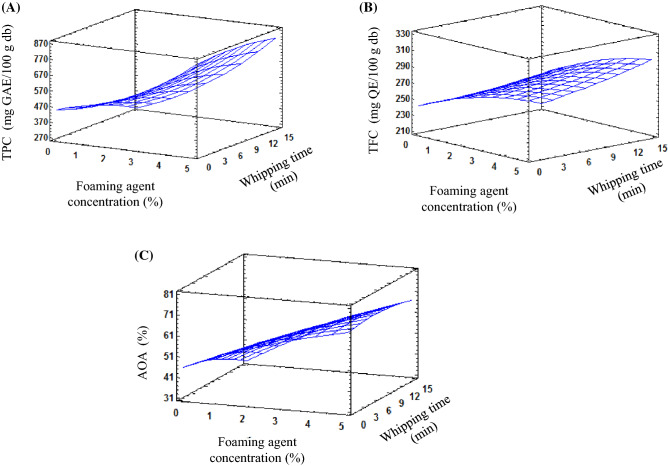
Response surface graph for phytochemical properties of hot air FMD tomato powder prepared with different concentrations of 32% SPI solution during different times of whipping: (**A**) total phenolic content (mg GAE/100 g db); (**B**) total flavonoid content (mg QE/100 g db); (**C**) antioxidant activity (%)

The contents of total phenolics (TPC) and total flavonoid (TFC) of HAD and FMD tomato powders are shown in Table 2. The TPC of HAD was found to be 422.3 mg GAE/100 g db, while that of FMD powders varied from 445.7 to 829.8 mg GAE/100 g db, with an increase of about 97%, for the powder prepared under foaming conditions of *C* = 5% and *t* = 8 min, compared to HAD powder. As to TFC, the TFC of HAD powder was 227.3 mg QE/100 g db, while that of FMD powder ranged from 252.4 to 316.6 mg QE/100 g db, with an increase of 39% for the powder prepared under foaming conditions of *C* = 5% and *t* = 8 min, compared to HAD powder. The lower levels of TPC and TFC of HAD powder may be due to the thermal degradation of these bioactive compounds (Brar et al., 2020) as well as the enzymatic oxidation by polyphenol oxidase which in turn alters the structure of the polyphenols (Lim and Murtijaya, 2007) as a result of long drying time. The impact of foaming parameters (*C*, *t*) on TPC and TFC is shown in Fig. 3. The TPC and TPC showed an increasing tendency with an increase in the concentration of SPI, while a contradicted behavior was observed with the whipping time. The increase in TPC and TFC may be due to (1) the retention of original bioactive compounds of tomato owing to the short time of FMD (Shaari et al., 2018), (2) the release of bound phenolics as a result of cell rupture during the puree preparation and whipping processes increasing the free phenolics, (3) the inherent phenolic content of SPI and (4) the pH influence; slightly acidic pH (pH of SPI solution: 6.8) could increase the release of bound TPC and TFC (Settharaksa et al., 2012). Therefore, the increase in TPC and TFC of FMD powders is a net result of a combined increase in their extractability (the original phenolics and bound phenolics released) and the inherent phenolics of SPI. On the other hand, the effect of whipping time could be explained by the fact that an excessive whipping may lead to a decrease in the TPC and TFC as a result of the oxidation of these compounds by the incorporated air into the foam structure, and to their thermal degradation by heat generated during the long whipping time (an experimental observation). Similar trends were obtained by Shaari et al. (2018) who reported an increase in TPC of FMD pineapple powder with an increase in the concentration of egg white. However, contradicted results were observed by Vasudevan et al. (2020) who found a decrease in the TPC of FMD soursop powder with an increase in the concentration of gum Arabic and fish gelatin, while Kadam et al. (2012) reported an insignificant effect of the foaming agent on the TPC of FMD pineapple powder.

### DPPH radical scavenging

The antioxidant properties of food materials are related to the presence of bioactive compounds in high amounts. The antioxidant activity (AOA) of HAD powder was found to be 43.9%, while that of FMD powder ranged from 47.3% to 71.1%, with an increase of 62% for the powder prepared under foaming conditions of C = 5% and t = 8 min, compared to HAD powder (Table 2). The obtained results are in the range of reported results where Hossain et al. (2021) found that AOA of FMD tomato powder prepared with egg white ranged from 51.6% to 71.42%. Similar trends were observed by Lobo et al. (2017) who found that FMD mango prepared with soy lecithin and carboxy methylcellulose had higher AOA than those of the control sample. The lower AOA of HAD powder may be related to the low levels of phenolic compounds (TPC) and flavonoids (TFC). The effect of foaming parameters (*C*, *t*) on the AOA is illustrated in Fig. 3. The AOA of FMD powder increased with an increase in SPI concentration, while it decreased with an increase in the whipping time. The higher antioxidant properties of FMD tomato powder could be related to the higher content of TPC and TFC. The reasons beyond the increase in AOA of FMD tomato powder are the same reasons for the increase in both the TPC and TFC as previously discussed. Padmaja and Srinivasulu (2016) reported that Ocimum sanctum leaves extract showed a high antioxidant capacity at pH 6.06, which decreased with an increase in pH to 7.6.

### Correlation matrix

A correlation matrix was performed to study the relationship between foaming parameters (*C*, *t*) and the response variables (Table 3). These correlations showed the predominant effect of SPI concentration on the physical and phytochemical properties of FMD tomato powder compared to the whipping time. This may be due to the close limits of studied whipping time. Some correlations were obtained between the concentration of SPI and response variables as well as between the response variables each to other as follows: (1) a strong negative correlation (− 0.91) was observed between the concentration of SPI and the bulk density of FMD powder. This correlation reflects the good foaming properties of SPI as a foaming agent and its capacity to produce a powder with higher porosity and less dense structure, (2) strong positive correlations were obtained between the concentration of SPI and each of the intrinsic density (0.99), relative expansion ratio (0.92), porosity (0.95), TPC (0.96), TFC (0.94), and AOA (0.95). These correlations reflect the chemical and textural changes that occurred during the FMD process such as the contribution of SPI to an increase in the total solids, TPC, and TFC of the powder. Moreover, these correlations reflect the rupture of cell walls releasing the matrix-bound phenolic compounds which in turn contributes to an increase in the AOA, and (3) strong negative correlations were observed between the bulk density and each of the porosity (− 0.99), relative expansion ratio (− 0.99), and intrinsic density (− 0.94). These correlations reflect the porous structure of the powder that resulted from the presence of the interstitial and occluded air between and within the particles.

**Table 3:** Correlation matrix between foaming parameters (*C, t*) and various response variables; physical and phytochemical properties, of hot air FMD tomato powder prepared with different concentrations of SPI during different times of whipping

Corr. Coef	C	t	ρ_bulk_	ρ_int_	ε_rel_	Porosity	TPC	TFC	AOA
C	1	0	− 0.91	0.99	0.92	0.95	0.96	0.94	0.95
t	0	1	− 0.24	0.01	0.23	0.15	− 0.17	− 0.22	− 0.27
ρ_bulk_	− 0.91	− 0.24	1	− 0.94	− 0.99	− 0.99	− 0.82	− 0.81	− 0.80
ρ_int_	0.99	0.01	− 0.94	1	0.94	0.97	0.95	0.92	0.92
ε_rel_	0.92	0.23	− 0.99	0.94	1	0.99	0.85	0.82	0.80
Porosity	0.95	0.15	− 0.99	0.97	0.99	1	0.87	0.86	0.86
TPC	0.96	− 0.17	− 0.82	0.95	0.85	0.87	1	0.95	0.94
TFC	0.94	− 0.22	− 0.81	0.92	0.82	0.86	0.95	1	0.98
AOA	0.95	− 0.27	− 0.80	0.92	0.80	0.86	0.94	0.98	1

*FMD* Foam-mat-dried tomato powder, *C* concentration of SPI (%), *t* whipping time (min), *ρ*_*bulk*_ bulk density (Kg/m^3^), *ρ*_*int*_ intrinsic density (Kg/m^3^), *ε*_*rel*_ relative expansion ratio (%), *TPC* total phenolic content (mg GAE/100 g db), *TFC* total flavonoid content (mg QE/100 g db), *AOA* antioxidant activity (%)

This study showed that the concentration of SPI was the predominant parameter affecting the physical and phytochemical characteristics of FMD tomato powder. The foaming conditions of 5% as a concentration of SPI and 8 min as a whipping time allowed to produce a tomato powder with a porous structure and improved physical and phytochemical properties. This study also stated the promising use of SPI as a foaming agent and its role in increasing the content of bioactive compounds and antioxidant activity of tomato powder. Due to the high content of phytochemicals having high antioxidant properties, the FMD tomato powder prepared with SPI could be used as a functional food in many dishes such as tomato soup and cooked dishes.

## Abbreviations

AOA: Antioxidant activity (%)
C: Concentration of soy protein isolate (%)
FMD: Hot air foam-mat drying
HAD: Conventional hot air drying
ρ_bulk_: Bulk density (Kg/m^3^)
ρ_int_: Intrinsic density (Kg/m^3^)
RSM: Response surface methodology
SPI: Soy protein isolate powder
t: Whipping time (min)
TFC: Total flavonoid content (mg QE/100 g db)
TPC: Total phenolic content (mg GAE/100 g db)
